# Development of an international Core Outcome Set (COS) for best care for the dying person: study protocol

**DOI:** 10.1186/s12904-020-00654-6

**Published:** 2020-11-30

**Authors:** Sofia C. Zambrano, Dagny Faksvåg Haugen, Agnes van der Heide, Vilma A. Tripodoro, John Ellershaw, Carl Johan Fürst, Raymond Voltz, Stephen Mason, María L. Daud, Gustavo De Simone, Kerstin Kremeike, Svandis Iris Halfdanardottir, Valgerdur Sigurdardottir, Jeremy Johnson, Simon Allan, Haroon Hafeez, Catarina Simões, Katrin Ruth Sigurdardottir, Birgit H. Rasmussen, Paula Williamson, Steffen Eychmüller, in collaboration with the iLIVE consortium, A. van der Heide, E. Geijteman, L. van Zuylen, K. van der Rijt, I. Korfage, A. Goossensen, H. van der Kuy, B. Yildiz, E. Elsten, J. Ellershaw, R. M. Smeding, S. Mason, T. Mc Glinchey, R. Hughes, R. Voltz, J. Strupp, M. Joshi, C. J. Furst, B. Rasmussen, M. Schelin, U. Lunder, H. Kodba-Čeh, M. Bakan, S. Eychmüller, S. C. Zambrano, N. Lüthi, M. Egloff, A. Christen, M. Martin Rosello, P. Barnestein Fonseca, I. Ruiz, D. R. F. Haugen, K. Sigurdardottir, K. Solvag, G. Skorpen Iversen, V. Sigurdardottir, S. I. Halfdánardottir, V. Tripodoro, A. Goossensen, J. Simon, C. Fisher, M. Berger, S. Allan, M. Boughey

**Affiliations:** 1 University Center for Palliative Care, Department of Oncology, Inselspital, Bern University Hospital, Bern, Switzerland; 2 University of Bergen and Haukeland University Hospital, Bergen, Norway; 3 Erasmus MC, Rotterdam, Netherlands; 4 Pallium Latinoamérica, Buenos Aires, Argentina; 5 Palliative Care Institute Liverpool, Liverpool, UK; 6 The Institute of Palliative Care, Lund University, Lund, Sweden; 7 Department of Palliative Care, Universitätsklinikum Köln (AöR), Köln, Germany; 8 Palliative Care Unit, Landspitali-National University Hospital, Reykjavik, Iceland; 9 Karunashraya, Bangalore, India; 10 Arohanui Hospice, Palmerston North, New Zealand; 11 Shaukat Khanum Memorial Cancer Hospital & Research Centre, Peshawar, Pakistan; 12 Palliative Care Team H. Luz Arrábida, Vila Nova de Gaia, Portugal; 13 University of Liverpool, Liverpool, UK

**Keywords:** Core outcomes set, Outcomes, Outcomes research, Delphi study, Palliative care, Last days of life, End of life

## Abstract

**Background:**

In contrast to typical measures employed to assess outcomes in healthcare such as mortality or recovery rates, it is difficult to define which specific outcomes of care are the most important in caring for dying individuals. Despite a variety of tools employed to assess different dimensions of palliative care, there is no consensus on a set of core outcomes to be measured in the last days of life.

In order to optimise decision making in clinical practice and comparability of interventional studies, we aim to identify and propose a set of core outcomes for the care of the dying person.

**Methods:**

Following the COMET initiative approach, the proposed study will proceed through four stages to develop a set of core outcomes: In stage 1, a systematic review of the literature will identify outcomes measured in existing peer reviewed literature, as well as outcomes derived through qualitative studies. Grey literature, will also be included. Stage 2 will allow for the identification and determination of patient and proxy defined outcomes of care at the end of life via quantitative and qualitative methods at an international level. In stage 3, from a list of salient outcomes identified through stages 1 and 2, international experts, family members, patients, and patient advocates will be asked to score the importance of the preselected outcomes through a Delphi process. Stage 4 consists of a face-to-face consensus meeting of international experts and patient/family representatives in order to define, endorse, and propose the final Core Outcomes Set.

**Discussion:**

Core Outcome Sets aim at promoting uniform assessment of care outcomes in clinical practice as well as research. If consistently employed, a robust set of core outcomes for the end of life, and specifically for the dying phase, defined by relevant stakeholders, can ultimately be translated into best care for the dying person. Patient care will be improved by allowing clinicians to choose effective and meaningful treatments, and research impact will be improved by employing internationally agreed clinically relevant endpoints and enabling accurate comparison between studies in systematic reviews and/or in meta-analyses.

## Background

In contrast to the typical measures employed to assess healthcare outcomes such as mortality or recovery rates, difficulties exist in defining which specific outcomes of care are the most important when caring for dying individuals. In the last phase of life, a variety of dimensions of care have been found to be important, such as symptom control, whole person care, quality of death and dying, and a good death [1]. A focus on these constructs as care outcomes has led to the development of interventions and measures that ensure that these standards are met, thus improving care at the end of life [2].

A main limitation of outcomes research in palliative care is that the focus of most interventions or tools assessing goals, outcomes, and quality of care has been about end-of-life care or palliative care in general, and not for the last days of life. While some efforts have been made to specify distinct stages of palliative care: early palliative care, end of life care, and care of the dying, and assign to them a specific timeframe, no standardised definition/timeframe exists [3–5]. Yet, the last days of life, or ‘care of the dying’, is considered as a discrete phase of palliative care [6]. The identification and understanding of needs and concerns in the last days of life is important to inform patient care because as patients’ health status changes, e.g. due to an acute deterioration, they may become more aware of their impending death, and change their own goals of care (such as those identified in Advance Care Planning).

Similarly, another limitation of research focusing on outcomes of care is that it has been often produced from the perspective of health professionals and academics [7]. Patients and families have been given less opportunities to define which outcomes are of importance to them. With their medical background, health professionals may give more relevance to medical aspects of care, while for the patient, there may be other pressing issues [7]. As an example, of the many areas integral to the provision of palliative care, the majority of measures have been found to over-represent physical symptoms, and to fail to address the care needs of the imminently dying patient in cancer and non-malignant advanced illnesses [6, 8, 9].

Questions regarding patient priorities as to which outcomes of care are more relevant in the last days of life remain relatively unexplored, or lacking direct assessment of patients in the last days of life [10]. Given that so many patients access palliative care at a late stage, and many other patients never receive palliative care [11], how can services best identify and meet the needs and goals of dying people, and how can they effectively measure them? There is a pressing need to undertake research with a more narrow focus into this stage [12]. During the last days of life, the symptoms of dying, as well as the illness itself and the awareness of dying may affect the patient at a level in which it had not before, and many areas of patient and family need could be neglected, or given less importance based on current outcome standards [13]. What has been overlooked is the possibility that the patients’ wishes may evolve during the course of the illness and the treatment offered during the last days of life may not be in line with their wishes. Capturing patient preferences at the right time also has the potential to produce meaningful results that will be of relevance to their own needs: “*the issues that are important to the dying are not likely to be the same at all stages of their illness: the problems of patients very close to death need to be dealt with on the basis of evidence obtained in patients very close to death*” [14].

With the shift towards patient autonomy and person-centred care, the patient perspective has become a predominant voice in the assessment of the quality of care, and more importantly, in the establishment of goals and outcomes of care [7, 15–17]. Outcomes research is leading health care professionals to recognize that it is the patient who determines the success of medical treatments, both when measuring outcomes and when defining them [15, 18–20]. While patient-centred outcomes have traditionally been of importance in palliative care research and education, the patient perspective in the definition of outcomes has been less studied, leading researchers to question themselves on whether current models of service delivery and research agendas do reflect patient priorities [13].

Originally intended for the measurement of the effects of specific interventions in clinical trials, core outcomes of care are increasingly being employed outside the research context to ensure that patient care is relevant and so that it can be efficiently measured and compared across services [17].

In palliative care, outcomes research has gained an important role in defining the need, value, and quality of care at the end of life [20]. However, outcome heterogeneity has been identified as one of the reasons why palliative care research has failed to inform clinical practice [21–23]. National registries of palliative care outcomes have been implemented in many countries as a quality improvement strategy. Among these, the Swedish Palliative Care Registry is one of the few to include measures of the quality of care relevant to the last week of life [24].

To date, despite efforts to harmonise *how* palliative care outcomes should be measured (i.e. through which tools or instruments) [25], no international consensus process has been undertaken to determine ***what*** the main outcomes of care are, (i.e. what dimensions should be addressed as the outcomes of care) particularly for the last days of life and including the patient perspective.

### Development of a Core outcome set (COS) for the last days of life

The Core Outcome Measures in Effectiveness Trials (COMET), an international initiative initially funded within the European Commission 7th Framework Programme, became a main advocate of outcome standardisation through the development of Core Outcome Sets. A COS represents a consensually predefined minimum of important outcomes that should be measured in a specific context. COS are increasingly being developed and implemented in several areas to ensure comparability across studies and clinical services, and to reduce research waste due to outcome heterogeneity [26, 27]. While there is an expectation and motivation to employ them as a predefined minimum in all trials and also to compare quality of care across clinical services, their utilisation does not restrict the inclusion of other outcomes that may be of interest and which may not be part of the minimum; in fact, as long as they are included, outcomes in a COS do not need to be a primary outcome [28]. This means that researchers will not have to exclude outcomes of their own interest, but that by employing the COS, the findings of their research will have a greater impact.

The COMET initiative centralises a registry of COS development studies to increase collaboration between researchers and to avoid duplication of COS. On their website, there are no groups focusing on COS development for palliative care in general, nor for the last days of life. Of the registered studies relevant to palliative care, one is a systematic review describing the range and quality of outcome measures in care homes [29], and another one is a position paper about the need to harmonise outcome measures in palliative care [30]. The only COS development projects in palliative care are focused on: family bereavement following an illness [31], treatment of psychological distress for family caregivers of patients with cancer or in palliative care [32], outcomes for lung cancer [33], COS measures for colorectal cancer [34], and a COS for the prevention of delirium [35].

### Scope of the COS

This project is embedded as a Work Package into a larger project funded by the European Union’s Horizon 2020 research and innovation programme, entitled iLIVE, which follows the research activities of the International Collaborative for Best Care for the Dying Person (derived from the former OPCARE 9 project, EU 7th Framework). This will be the first study to investigate and define *what* the core outcomes for the last days of life are, and to include in the consensus process the perspective of families, patient representatives, and other key stakeholders. Taking into consideration that within palliative care the family and the patient form the unit of care [36] and that outcomes of the last days of life impact family wellbeing after death, the COS will be developed to include outcomes relevant to the patient and the family. Although most COS focus on outcomes relevant to patients only, examples of COS including two target groups exist. For example, a COS for the prevention of preterm birth endorsed a final COS that included items for the pregnant women, as well as outcomes focused on the offspring [37].

This project aims to identify and propose a set of core outcomes for best care for the dying person, where dying is understood as the point at which individuals are clinically recognised to have entered the dying phase and may thus have only hours or days to live, independent of their diagnosis, grounded in a systematic review of the literature and determined through the perspective of patients, family members, researchers, and health professionals. The COS is intended to be used for research purposes, as well as in clinical practice, that is, it will optimise both decision making in clinical practice, as well as improve the comparability of interventional studies. The COS is expected to be relevant in all settings where an individual may be cared for at the end of life: at home, in the hospital, in hospices, or in other facilities.

## Methods/design

**Fig. 1:**
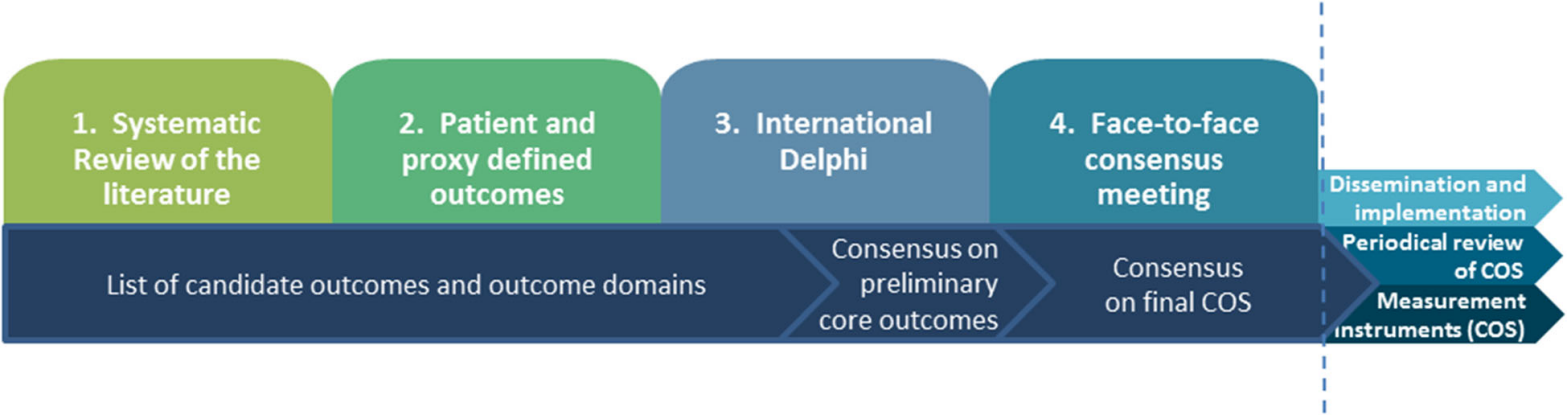
Study design

The study will follow the approach suggested by the COMET (Core Outcome Measures in Effectiveness Trials) initiative [38, 39] and will adhere to the COS-STAD recommendations for COS development [40]. The development of COS involves four stages: 1) a systematic review to identify currently employed outcomes, 2) studies to identify the patient and family perspective, 3) a Delphi study to prioritise outcomes derived from stages 1 and 2, and 4) a face-to-face consensus meeting to agree and endorse the final COS (see Fig. 1). Through this process we will define what are the important outcomes of care for the last days of life. Recommendations on how to assess the outcomes will be provided on the basis of the systematic review, as well as through existing recommendations such as those developed by MOREcare [25]. The COS development study has been registered on the COMET database (http://www.comet-initiative.org/studies/details/967).

### Stage 1: systematic review

A systematic review is one of the main steps in the identification of existing outcomes. The review aims to summarize what outcomes are systematically measured at the end of life, how are they assessed, that is, through which specific tools, when are they assessed, who reports on the outcome: whether the patient, the family or the health professionals, and which of these outcomes would be relevant for the last days of life.

#### Definitions

For the purpose of the systematic review, we need to predefine two central concepts: *outcomes*, and *end of life*. Considering the specific features and domains of palliative care, outcomes will be understood as the effect or the end result of a given treatment/intervention on the patient, the family of the patient, the medical services, or on the health system. Although the COS will focus on patient and family outcomes, the review will take into account all other outcomes assessed in the studies to be included. Outcomes to be extracted from the studies will include not only those strictly denoted as ‘outcomes’ or ‘endpoints’, but particularly in the observational studies, they will be identified in terms of specific areas of interest such as: ‘satisfaction with care’, ‘quality indicators; e.g. ‘quality of life’, ‘quality of end of life’, ‘quality of death and dying’, ‘perceptions’, ‘standards’, ‘goals of care’, ‘end of life wishes’, or ‘factors considered important in the last days of life’.

Taking into account the lack of a standard definition or a specific time frame for the term *end of life* [4, 41] we will include literature focused on people with incurable, advanced illnesses who are considered to be at the end of life, but with an explicit life expectancy of *one month* to *days* of life, so that the outcomes identified are as close as possible to the dying phase. To narrow down the search terms for ‘end of life’ we will include a combination of those employed by the National Institute for Health and Care Excellence in their development of the National Guidelines for the Care of Dying Adults in the Last Days of Life [42]. All outcomes from these studies will be selected for the long list to be generated for the Delphi Study (Stage 3). Studies which do not specify or which involve a variety of periods will not be included for review.

#### Types of studies

The review will focus on outcomes which have been employed in interventional studies, as well as outcomes identified through other peer-reviewed literature, including observational studies, surveys, and qualitative studies. Grey literature, patient registries, and relevant national guidelines or quality improvement initiatives from different countries will be searched for and included in the analysis when relevant. Outcomes from ongoing trials registered in the International Clinical Trials Registry Platform (ICTRP) from the World Health Organisation [43] (which includes data from at least 17 independent international registries) will be extracted. The protocol for the systematic review has been registered prospectively on the PROSPERO database (registration number pending).

#### Types of participants/population

This review will consider studies if they include adults who were at the *end of life.* However, since the term has a broad definition of a life expectancy of months to days of life, we will narrow our selection to include only those studies reporting no longer than the last month of life. Studies will be considered if outcomes are defined or reported by patients, health professionals, and/or family members. Outcomes will be included whether they directly affected the patient, the family members, the health professionals, or the health system as a whole. Studies undertaken in any setting of care (hospital, hospice, community, home) will be included.

#### Condition or domain being studied

The systematic review aims to identify outcomes which are considered important during the last days of life from the perspective of patients, family members, clinicians, and researchers. The outcomes can be identified in the context of any chronic illness or condition which is at an advanced stage such as advanced cancer, end-stage kidney, lung or heart disease, dementia, etc., and taking into account the variety of contexts where dying people can be cared for during the last days and weeks of life (e.g. home, hospital, hospice, aged care facilities).

#### Exclusion criteria

Studies will be excluded if they report on end of life aspects within paediatrics, on non-human research, or if they are from the perspective of healthy individuals or the general population, with the exception of bereaved family members and health professionals, as long as the time period considered in the specific study is limited to the last month of life.

#### Information sources

Studies will be retrieved from the following electronic databases: Medline, CINAHL, PsycInfo, and Embase. Unpublished interventional studies will be identified from the ICTRP and the Cochrane Central Register of Controlled Trials. In addition, a manual search of references from relevant articles will lead to identifying other potentially important publications. National guidelines, statements or standards will be searched for on the Internet and through a snowballing technique via the members of the Collaboration for Best Care for the Dying Person.

Studies in English, Spanish, and German will be considered for inclusion in the review. The search terms have been piloted and adapted to the syntax of all databases and have been uploaded to PROSPERO: International prospective register of systematic reviews.

#### Selection process

Initially, two review team members will screen the first 100 titles and abstracts independently and will compare their results. After checking for disagreements, titles and abstracts of all articles identified through the searches will continue to be screened independently by at least two researchers who will be blinded to each other’s decisions. In this initial screening process, duplicates will be discarded and each study will be categorized as: a) to be included, b) to be discarded, or c) unsure. A similar procedure will be followed with all excluded articles after full-text review. Full-texts of all potentially relevant articles, which fell in the ‘to be included’ or the ‘unsure’ categories, will be obtained and will be assessed according to the inclusion criteria. The process of article selection will be shown in a PRISMA flow diagram and the review will adhere to the PRISMA reporting guidelines [44].

At all stages, disagreements will be resolved through discussions until consensus is reached, and if there is no agreement between the reviewers a third party will be involved.

#### Data extraction process

From each included study a minimum of information will be extracted through a data extraction tool. The tool will be piloted and refined with an initial set of articles.

#### Risk of bias in individual studies

Since the main goal of the systematic review is to aid in the generation of the initial list of outcomes, the risk of bias of each of the studies, as well as the quality of each of the studies will not be assessed.

#### Strategy for data synthesis

A narrative synthesis of the findings of the review will be structured around the types of outcomes measured, the tools employed to measure these outcomes, the characteristics of the target population, and type of intervention (if it is an interventional study). If enough data are available, a subgroup narrative synthesis of differences between cancer and non-cancer patients, within non-malignant illnesses, differences according to age groups, place of care, and place of death will be made.

#### Selection of outcomes for Delphi study

Once outcomes have been identified, each individual outcome will be grouped into greater outcome domains. The taxonomy developed by COMET [45] will be employed. Depending on the length of the list, the outcomes will be grouped by frequency of use across the reviewed studies. A differentiation will be made between the outcomes identified in the interventional studies (most likely to be researcher-defined), and those arising from other types of studies (most likely to be participant-defined –that is from the user perspective).

### Stage 2: definition of patient and proxy defined outcomes

A second stage will determine patient and proxy defined outcomes of care important to the last days of life. Within the iLIVE project, data on outcomes relevant to patients and families will be collected prospectively, and longitudinally, as part of a cohort study in 11 countries (Netherlands, UK, Germany, Sweden, Slovenia, Switzerland, Spain, Norway, Iceland, New Zealand, and Argentina). A separate protocol will describe this part of the study.

Studies about patient and proxy defined outcomes may also be duplicated in several countries, under the lead of members of the International Collaborative for Best Care for the Dying Person. The aim of these studies will be to define, from the perspective of the patients and bereaved relatives, what are the aspects and goals of care which are most relevant in the last days of life. In addition, outcomes from ongoing or prior studies undertaken by members of the collaborative, in which information about patient and proxy perspectives on outcomes relevant to the last days of life exists, will be included into the outcome list. Any potential ethical issues of re-analysing data collected for different purposes will be resolved before accessing the data (e.g. by anonymising datasets or seeking new ethical approvals).

The methodologies for studies to be included within this stage, outside of data collected through iLIVE, will be varied, e.g. in-depth interviews - particularly with family members, surveys, q-sort cards, or prospectively collected clinical data about expectations, goals and wishes of patients and family members. The specific methods will be chosen by each institution taking into account feasibility in each of the contexts/countries where the studies will be performed. Although having a variety of methods and sample sizes may be seen as compromising research rigour, since the outcomes to be extracted from these studies will populate the greater outcome list to be used at the Delphi stage, we believe that this is not a limitation of our study, as outcomes will not be weighed on the number of participants who mention them.

Research efforts will take into account individual, social and cultural differences. This will be crucial in producing results which can help health professionals to identify, not only the core outcomes as defined by the participants themselves, but also, to devise and identify potential measures to assess and identify whether these outcomes are being achieved.

All studies will obtain local ethical approval prior to study start. The Swiss study about patient and family defined outcomes was given clearance by the local ethics committee (KEK 2016–00896).

#### Participants

##### Patients

The main target group for data collection is of individuals who can be characterized as living their last days of life (7 days before death). However, taking into consideration the difficulties in recognising dying [46, 47], and the difficulties of obtaining patient consent at this stage, within iLIVE, we aim to collect this data longitudinally with patients who have a life expectancy of 6 months or less and who will be followed up 1-month after study inclusion. While some participants would not be as close to living the last days of life as others, all participant data will nonetheless be employed, and aspects for potential comparisons, differences and similarities between groups will be considered. Potential patients should have the ability to provide informed consent (if required for the specific type of study), be 18 years old or older, communicate in the language in which the research will be undertaken, and be able to tolerate the demands of participation depending on the study format.

##### Family members

The perspective of family members on what outcomes are important, for them as individuals, and for the dying family member in the last days of life can be collected prospectively or retrospectively. Prospective data within iLIVE will be collected from families starting from a life expectancy of 6 months or less, 1 month after study inclusion, and about 3 months after death. In studies where data is collected retrospectively, bereaved family members will be invited to participate in the study within 3 to 12 months from the death of their family member. While different time frames have been used in bereavement studies, and a potential optimal time frame has been found to vary between individuals [48], we have selected 3–12 months after death, because this timeframe may be seen more positively by the stricter ethics committees, while still being early enough to facilitate recollection of what happened during the last days/weeks of life of a dying family member. Participants must be able to provide informed consent and must speak fluently in any of the languages of the study. Prospective studies will focus on the experiences as they are occurring to family members before death.

#### Data collection

Data collection and sampling will be undertaken as defined in each study protocol. In each of the participating countries, particularly in those where a qualitative design will be employed, data from patients and bereaved relatives is expected to be collected in parallel, in order to enrich the data collection process.

Outcome data from all international studies will be collated and added to the list of outcomes identified through the systematic review of the literature. Any outcome ambiguities will be resolved with the Leadership group for COS development (composed by a smaller group of clinicians and researchers involved in the studies, and lay people or their representatives).

#### Data analyses

Data will be analysed according to the specific methodological approach, e.g., qualitative methods for interview data, while survey or Q-sort card data will be analysed through descriptive statistical techniques, or a mix of quantitative and qualitative methods, as will be the case with the data collected prospectively through iLIVE. Ultimately, each individual project leader will define the procedure, as long as they can provide the COS development team results that can be incorporated into the outcomes list and that have been developed following the required local ethical requirements.

#### Identification of main outcomes

This stage of the COS development process aims to provide a greater understanding of the priorities and needs of patients and families during the last days of life and will shed light on whether patient and family priorities for the last days of life are different from those currently employed as outcome measures. The studies on the patient and family perspective will help identify a list of outcomes of importance to each of these groups in the last days of life.

Taking into account the intended heterogeneity of the sample (differences in time to death, ages, genders, diagnoses, and cultural backgrounds) the analyses will take into account these potential subgroups to identify whether differences may exist in the end of life preferences/outcomes of each group. Differences in outcomes that are important for family members in contrast to those of patients will also be noted, and if different, they will justify the development of outcomes focused on the patient, and outcomes focused on the family.

### Stage 3: international Delphi study

The Delphi study has been prospectively authorised by the Bernese Cantonal Ethical Commission with a declaration of no objection, meaning that this part of the study is exempted from further ethical review (req-2019-00200). The potential long list of outcomes collated through stages 1 and 2 of the study, along with demographic items, will form the basis of the Delphi survey. Outcomes will be organised within domains. In order to reduce bias on the importance of each outcome, all outcomes will be presented in alphabetical order, per greater domain and within each domain.

#### Participants

The four stakeholder groups of interest for the Delphi study will be clinicians, researchers, family members, and patient representatives. In stages 3 and 4 the inclusion of patient participants will not be possible since participants’ illnesses will be far too advanced to enable them to comply with the demands of the Delphi study or to attend the consensus meeting. One of the main challenges of COS studies is to preserve the patient perspective throughout the Delphi study and the consensus meeting, particularly when they are not able to participate actively. Therefore, in this study we will include a patient representative group formed by people heavily involved in providing non-professional support at the end of life, such as volunteers. Palliative care volunteers have close proximity to patients up to late stages of the illness, including the moment of death, and therefore can speak about their needs in a way that would be more difficult for family members. Public involvement associations and organisations will be contacted to support this task. In the countries where no formal volunteer services exist, local patient representative organisations will be asked to nominate suitable participants to represent the views of patients.

While the specific number of participants per group will not make a difference for analysis, since we will present results per group, considering the importance of each of the four stakeholder groups, we expect that all participant groups have a similar number of participants. To reduce attrition, measures such as acknowledging participation of each individual in all publications will be employed, as long as the individual agrees for their name to be published.

According to COMET guidelines for the development of COS, a sample size calculation for Delphi studies is not required and is not dependent on statistical notions such as power, as it is often one of convenience [39]. To increase uptake and value of the COS, however, it is crucial that the Delphi participants can be described as experts within their stakeholder groups. In addition, ensuring representation from a variety of countries and different demographic characteristics across and within groups brings more value by making the COS more internationally relevant and thus more generalisable. Therefore, as a minimum, we will include participants from the eleven countries participating in the cohort study within the iLIVE project, ensuring international heterogeneity and relevance. From each country we will obtain a list of 10 potential participants per stakeholder group. Taking into consideration that there are 4 stakeholder groups, 40 individuals will be contacted per country, for a total of 440 potential participants. Equal numbers per country, per stakeholder group will not be a requirement, as long as the countries have similar characteristics, e.g. similar global location, cultural and spiritual characteristics such as religiosity. This means that countries such as Argentina and New Zealand, for example, should not be merged in the same group. Should groups need to be merged, the decision will be made by the COS Leadership group. In addition, outside of the countries participating in iLIVE, which already represent South America, Europe, and Oceania, we will have representation from Portugal, India and Pakistan, and through the contacts from the International Collaborative for Best Care for the Dying Person, we will extend the invitation to the different members, who represent about 21 countries of different income levels and regions. Other international associations will be asked to support recruitment for the Delphi study, according to need.

##### Clinicians

Physicians, nurses and allied health professionals dealing with patients at the end of life, including palliative care specialists, mobile care teams, community nurses, general practitioners, but also those practising in other settings where people die, such as the emergency department, the intensive care unit, and aged care facilities, etc., will be invited. Each of the countries will nominate key people from their own countries, but also at an international level, so as to ensure wider international relevance of the COS.

##### Researchers

Active researchers will be identified by each of the participating COS development groups. Similar to the selection criteria for clinicians, it is expected that this group is heterogeneous in terms of main degree, years of practice, seniority, etc. These differences will help ensure representativeness.

##### Patients

Direct participation of patients will not be possible. With the specific timeframe of the study being the last days and weeks of life, participants will not be able to participate in the Delphi study. Therefore, their voice will be heard in stages 3 and 4 through people who have contact with patients during this time, and who can speak for them without letting their own personal needs come in the way. We consider hospice and palliative care volunteers to be an ideal group to voice the needs of patients in the last week of life. Opposite to family members, who can only speak from their individual experience, volunteers have generally wide experience with patients and can thus act as advocates for the patient group as a whole.

##### Family members

Family members from deceased patients will be identified through the centres associated with project members of the iLIVE project. These include hospitals, hospices, and palliative care units. We will include family members who already have experienced the death of a significant other. Because we are focused on the last week of life, we do not want to ask families when it is too early for them to imagine what the needs in the last week of life will be like, nor closer to the death of their significant other death, as that time is intensely personal.

In order to facilitate participation of individuals with a non-academic/non-scientific background, they will be provided with definitions, explanations, and clear instructions through written means, and they will also be given the details of a local contact person who can solve any unanticipated issues, if any arise. All participants will be initially contacted electronically and invited to participate in the study. However, if the electronic format was too difficult to implement with a particular stakeholder group, printed versions of the Delphi questionnaire will be sent by post and reply paid envelopes will be provided to them. Depending on the specific round, participants will also receive prior results in this same format. Their answers will later on be transcribed into the *DelphiManager* system so that they can be computed along with all other responses.

#### Delphi rounds

**Fig. 2:**
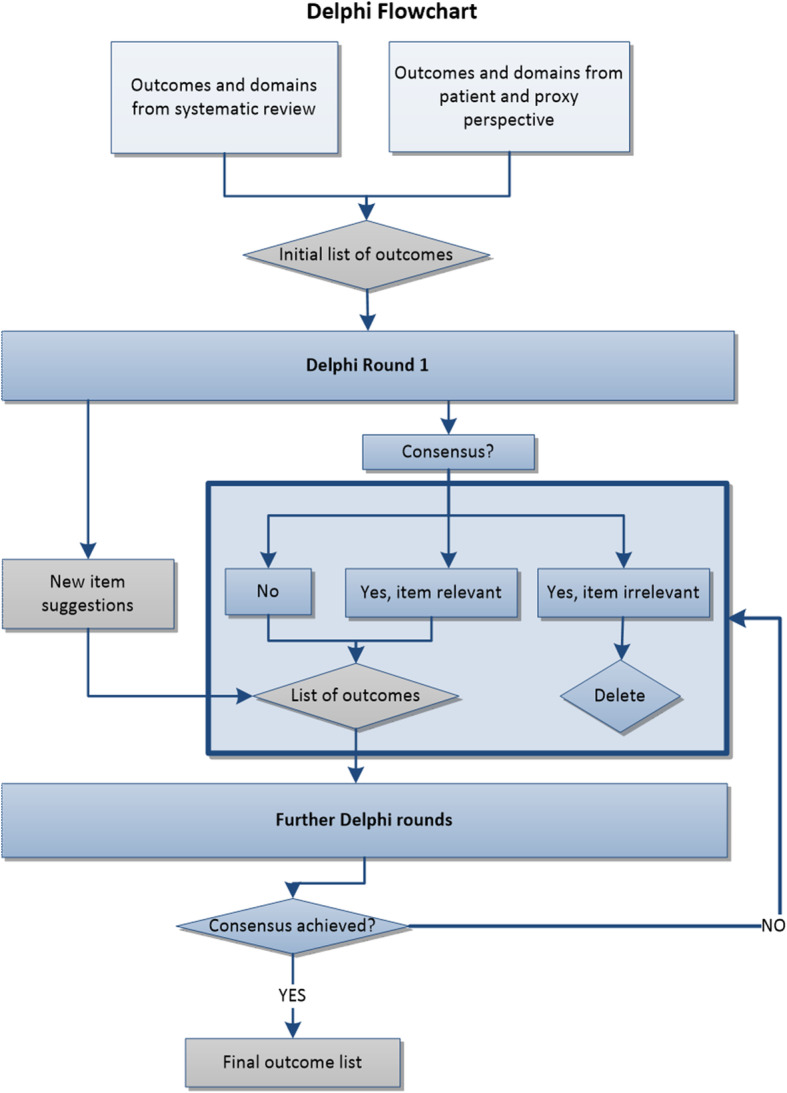
Delphi study flowchart

The *DelphiManager* system from the COMET initiative will be employed to build, distribute, and manage the Delphi surveys. The Delphi study will consist of at least two rounds, with the final number of rounds determined on whether consensus was reached according to pre-specified criteria (see Fig. 2).

In Round 1, all preselected outcomes will be presented in a list and each participant will be asked to rank each one of them from 1 to 9, where 1 is the least important and 9 is of highest importance. Following the scale from the GRADE group [49], the lowest scores (1, 2, and 3) will be considered as ‘*not that important*’, 4 to 6 will be considered as ‘*important but not critical’*, and 7 to 9 will be considered as *‘important and critical’*. To determine which items should be in the final list, the thresholds suggested by the COMET group [38] will be employed: per item, *consensus to include* an item will be when over 70% of the participants score the item between 7 to 9, and less than 15% of the participants scored the same item as 1 to 3. *Consensus to exclude* an item, on the other hand, will be achieved when over 70% of the participants have scored the item between 1 to 3, and less than 15% of the participants score the same item between 7 to 9. Any uncertainties in establishing consensus will be discussed and resolved with the COS Leadership Group.

Participants will be given 3 weeks to respond the survey and will receive a reminder at the beginning of the second week. In this same round, participants will be asked to nominate any outcomes which are relevant from their perspective, but which were not included in the initial list. This will facilitate inclusion of outcomes that may be relevant but that we did not obtain in stages 1 and 2 of the study.

Round 2 and round 3 will include all items selected through the *consensus to include* procedure, as well as those where there was no consensus, and all new items proposed by the participants (new items will only be asked for in the first round, therefore no new items will be added beyond Round 2). Items for which *consensus to exclude* was reached, will be omitted. In Round 2 and any subsequent rounds, participants will be given the opportunity to see a histogram displaying the distribution of scores given by participants from each stakeholder group, along with their own previous score, and will be able to re-score each item if they wanted to.

Participants will receive four sets of results, one from each of the stakeholder groups separately. This means that each individual participant will see the scores given by the clinician group, the researchers, the patients (through representatives), and the family members. Next to these would be their own previous score and they would have the opportunity to change or reinstate their score.

If after three rounds several items still remain without consensus, the Delphi study will be terminated and the outcomes where no consensus was found, will be presented at the consensus meeting. An effort will be made to ensure a similar number of participants per group, so as to not give more influence to a particular group. When differences exist between stakeholder groups in the final list of core outcomes, these differences will be preserved and discussed during the consensus meeting. That is, if there was no final consensus across the four stakeholder groups, four sets of outcomes will be presented at the consensus meeting.

### Study 4: face-to-face consensus meeting

In the development of a COS, the final step is a face-to-face consensus meeting. The main outcome of the meeting will be the definition, endorsement, and proposal of the final core outcome set, including any potential domains and subdomains. The Bernese Cantonal Ethical Commission authorised the consensus meeting declaring it exempt from further ethical review (req-2019-00200). The consensus meeting is expected to take place as an independent event, possibly within the context of an international meeting dedicated to the discussion of end of life outcomes and organised by the COS development group. This way, before participants are shown the results of the Delphi, they can be socialised to different perspectives on outcomes relevant at the end of life, which may help them decide their votes. Participants for the consensus meeting will be purposefully selected from those participating in the Delphi study, to ensure representativeness from the greater group. Because of funding, not all countries will be expected to have representatives from the 4 stakeholder groups. From the researcher and clinician groups, it is expected that at least all countries are represented by one of each group (20 participants), for the family and patient representatives, at least half of this number will be expected (10 participants), and considering funding issues to cover their travelling fees not all countries are expected to have family or patient representatives present.

At the consensus meeting, all participants will be present in the same room and will be shown all the *consensus to exclude* items, as well as the results for the items where consensus for inclusion was reached, and those where no consensus was reached. To ensure that participants are not coerced, or have to vote under peer pressure for outcomes that they do not value, everyone will vote anonymously through tablets or their smartphones. Differences between participants will be negotiated and the final COS will be decided upon.

### COS dissemination and implementation plan

Beyond the development of a COS, COS developers are encouraged to devise strategies to improve the adoption of the COS in the settings that it has been devised for [39]. The COS for best care for the dying person is expected to have an impact in research and in clinical practice, therefore, the strategies to be implemented will address both contexts. Further to updating the COMET registry on the status of the COS, the COS development group will publish the final COS in a renowned palliative care journal, as well as aim to present it at an international palliative care conference. In addition, an editorial or commentary about the newly developed COS, as well as its expected impact in reducing research waste [26, 27] will be sought from a relevant palliative care journal.

A periodical review of COS uptake will help measure in which contexts and up to what extent the COS is being employed by clinicians and researchers. Through the periodical review, a decision on whether the COS may require an update to reflect newer trends or perspective may also need to be made, thus leading to repeating some stages of the COS development process.

## Discussion

The COS produced at the end of this study will identify ***what*** minimum outcomes should be employed in the last days/weeks of life, both in research and in clinical practice. A similar process to establish ***how*** to measure the proposed outcomes will be implemented after the development of the COS.

The development of core outcome sets is a healthcare priority. Without a unifying outcome framework through which the effectiveness of palliative care interventions can be compared across clinical trials in systematic reviews, research efforts will have little to no impact. If consistently employed, a robust set of core outcomes for the last days/weeks of life, defined by all relevant stakeholders, will ultimately be translated into best care for the dying person and their families. Patient care will be improved by allowing clinicians to choose effective and meaningful treatments, and the impact of research will be significantly improved by measuring generally agreed endpoints which can be compared between studies and in meta-analyses. The core outcome sets are expected to become standards of quality of the care provided to patients living the last days of life, and will also be standards for comparisons between clinical trials.

## Data Availability

This is a study protocol therefore no data has been generated that can be shared or made available to third parties.

## Abbreviations

COS: Core Outcome Sets
COMET: Core Outcome Measures in Effectiveness Trials
ICTRP: International Clinical Trials Registry Platform
